# Statement of Retraction

**DOI:** 10.1080/21655979.2026.2614847

**Published:** 2026-01-19

We, the Editor-in-Chief and Publisher of *Bioengineered (the “**Journal**”)*, have retracted the following articles which were published in the Special Issue titled “***International Conference on Sustainable Waste Treatment and Management (SWTM-2019)****”*, guest edited by Mukesh Kumar Awasthi, Binod Parameswaran & Zengqiang Zhang. *Bioengineered, 11(S11), originally published in 10(01) and 11-01*.

- Awasthi, M. K., Parameswaran, B., & Zhang, Z. (2019). The special issue on ‘International Conference on Sustainable Solid Waste Treatments and Managements (SWTM-2019).’ *Bioengineered, 11*(SI1), 697. https://doi.org/10.1080/21655979.2019.1703630
- Sarsaiya, S., Shi, J., & Chen, J. (2019). A comprehensive review on fungal endophytes and its dynamics on Orchidaceae plants: current research, challenges, and future possibilities. *Bioengineered, 11*(SI1), 316–334. https://doi.org/10.1080/21655979.2019.1644854
- Jain, A., Sarsaiya, S., Wu, Q., Shi, J., & Lu, Y. (2019). New insights and rethinking of cinnabar for chemical and its pharmacological dynamics. *Bioengineered, 11*(SI1), 353–364. https://doi.org/10.1080/21655979.2019.1652491
- Jain, A., Sarsaiya, S., Wu, Q., Lu, Y., & Shi, J. (2019). A review of plant leaf fungal diseases and its environment speciation. *Bioengineered, 11*(SI1), 409–424. https://doi.org/10.1080/21655979.2019.1649520
- Wainaina, S., Lukitawesa, Kumar Awasthi, M., & Taherzadeh, M. J. (2019). Bioengineering of anaerobic digestion for volatile fatty acids, hydrogen or methane production: A critical review. *Bioengineered, 11*(SI1), 437–458. https://doi.org/10.1080/21655979.2019.1673937
- Lukitawesa, Patinvoh, R. J., Millati, R., Sárvári-Horváth, I., & Taherzadeh, M. J. (2019). Factors influencing volatile fatty acids production from food wastes via anaerobic digestion. *Bioengineered, 11*(SI1), 39–52. https://doi.org/10.1080/21655979.2019.1703544

Following publication, the Publisher identified concerns regarding the editorial handling of the Special Issue and the peer review process.

An investigation by the Taylor & Francis Publishing Ethics & Integrity team in full cooperation with the Editor-in-Chief concluded that the articles included in this Special Issue were not peer-reviewed appropriately, in line with the Journal’s peer review standards and policy.

As the stringency of the peer review process is core to the integrity of the publication process, the Editor-in-Chief and Publisher have decided to retract all the articles within the above-named Special Issue. The Editor-in-Chief and the Publisher have not confirmed if all the authors were aware of the concerns with the peer review process.

The Journal is committed to correcting the scientific record and will fully cooperate with any institutional investigations into this matter. The authors have been informed of this decision.

We have been informed in our decision-making by our editorial policies and the COPE guidelines.

The retracted articles will remain online to maintain the scholarly record, but they will be digitally watermarked on each page as ‘Retracted’.

